# The Role of pH, Electrodes, Surfactants, and Electrolytes in Electrokinetic Remediation of Contaminated Soil

**DOI:** 10.3390/molecules27217381

**Published:** 2022-10-30

**Authors:** Brian Gidudu, Evans M. N. Chirwa

**Affiliations:** Water Utilisation and Environmental Engineering Division, Department of Chemical Engineering, University of Pretoria, Pretoria 0002, South Africa

**Keywords:** electroosmosis, electrophoresis, electromigration, biosurfactants, hydrocarbons

## Abstract

Electrokinetic remediation has, in recent years, shown great potential in remediating polluted environments. The technology can efficiently remove heavy metals, chlorophenols, polychlorinated biphenyls, phenols, trichloroethane, benzene, toluene, ethylbenzene, and xylene (BTEX) compounds and entire petroleum hydrocarbons. Electrokinetic remediation makes use of electrolysis, electroosmosis, electrophoresis, diffusion, and electromigration as the five fundamental processes in achieving decontamination of polluted environments. These five processes depend on pH swings, voltage, electrodes, and electrolytes used in the electrochemical system. To apply this technology at the field scale, it is necessary to pursue the design of effective processes with low environmental impact to meet global sustainability standards. It is, therefore, imperative to understand the roles of the fundamental processes and their interactions in achieving effective and sustainable electrokinetic remediation in order to identify cleaner alternative solutions. This paper presents an overview of different processes involved in electrokinetic remediation with a focus on the effect of pH, electrodes, surfactants, and electrolytes that are applied in the remediation of contaminated soil and how these can be combined with cleaner technologies or alternative additives to achieve sustainable electrokinetic remediation. The electrokinetic phenomenon is described, followed by an evaluation of the impact of pH, surfactants, voltage, electrodes, and electrolytes in achieving effective and sustainable remediation.

## 1. Introduction

Recently, electric and electromagnetic treatment methods, such as electrical resistance heating, radio frequency, microwave heating, and electrokinetic remediation, have caught the attention of several researchers [1,2]. The preference for electrochemical technologies is because of their low footprint and low production of wastes. In addition, they do not need auxiliary chemicals to be used and can be combined with other technologies to make remediation processes more efficient [3,4]. Electrokinetic remediation in particular has the potential to remediate media contaminated with chlorophenols, polychlorinated biphenyls, phenols, trichloroethane, BTEX compounds, and entire petroleum hydrocarbons [5,6,7]. Furthermore, unlike conventional remediation methods, electrokinetic remediation can be used effectively for both ex situ and in situ remediations of low permeability soils [8]. It can also be used in railway soils and residential areas where it could be difficult to excavate [8,9].

Compared to electrokinetic remediation, most conventional technologies for hydrocarbon containment and remediation, such as bioremediation, biostimulation, oil isolation and containment, bioventing, and most chemical treatment methods, are inefficient, introduce toxic compounds into the treated media, and may not necessarily eliminate the contaminant [10,11]. For instance, biological methods of remediation, such as bioremediation, bioventing, biosparging, bioaugmentation, biostimulation, and bioattenuation, have been reported to be inefficient and require long time periods to achieve substantive treatment [10], advanced oxidation chemical processes are associated with increasing soil acidity and producing more toxic products into treated media [12], while chemical treatment methods make use of organic compounds, such as EDTA, that are toxic and difficult to degrade [11,13]. On the other hand, adsorption methods, such as use of biochar, have shown great potential in removing hydrocarbons from soil and are environmentally friendly [14,15], but the long-term effects of biochar on soil mechanisms have not been determined under realistic conditions in field-scales studies [16].

In electrokinetic remediation, a current is applied across an electrode pair to induce movement of ions, charged particles, and fluids through a porous medium [6,17]. By employing electrochemical processes, such as electrolysis, electroosmosis, electrophoresis and electromigration, pollutants can be removed from contaminated media [7,18]. Some researchers have argued that it is almost impossible to obtain efficient electrokinetic remediation of contaminated soil by relying on only electroosmosis and electromigration [19]. Hence, electrokinetic remediation is improved by control of pH swings, use of highly ionic electrolytes, and use of either active or non-active electrodes in combination with other conventional remediation methods to improve the efficiency of remediation [20]. However, not all technologies, materials, additives, methods, and processes used to improve electrokinetic remediation are considered environmentally sustainable [21].

This article focuses on reviewing the impact of electrodes, pH swings, surfactants, electrolytes, and voltage and how these can be applied to achieve efficient electrokinetic remediation with a low environmental impact. Most previous review papers focused on the principles and applications of an electrokinetic system as a potential remediation technology for removing different contaminants, such as heavy metals and organochlorines. In this paper, the focus is on evaluating the effect of voltage, pH, electrolytes, surfactants, and electrodes on the fundamental processes of electrokinetics and how these can be altered or substituted with greener alternatives to attain sustainable electrokinetic remediation of hydrocarbon contaminated soil. The paper reviews the impact of voltage, pH, electrolytes, electrodes, and surfactants on electroosmosis, electromigration, and electrophoresis. Green alternative technologies or process agents that can be combined with conventional electrokinetic remediation are also proposed.

## 2. Electrokinetic Remediation

In the 19th century, the electrokinetic phenomenon was, for the first time, operated and observed by Reuss in application of current in clay–water media [22]. Helmholtz and Smoluchowski then conducted advanced studies that led to the identification of the likely processes and proposition of kinetic overviews [22]. In the application of electrokinetics for remediation, a current is applied across an electrode pair to induce movement of ions, charged particles, and fluids through a porous medium [23,24,25,26]. Depending on whether the method is used to remediate slurries, sludge, or soil, it may also be referred to as electrochemical decontamination of wastes, electrokinetic soil processing, or electro-reclamation [25,27,28].

Electrokinetic remediation has recently emerged as a method that effectively removes metals, anions, and polar organics from contaminated soils [29]. The compounds that have been studied using electrokinetic remediation in previous work include total petroleum hydrocarbons, trichloroethane, chlorophenols, toluene, polychlorinated biphenyls, phenols, BTEX compounds (such as ethylbenzene, benzene, toluene, and xylene), volatile organic compounds, lead, mercury, uranium, zinc, nickel, copper, chromium, cadmium, and arsenic [22,25,28,30].

### The Fundamental Theory of the Electrokinetic Phenomenon

**Figure 1:**
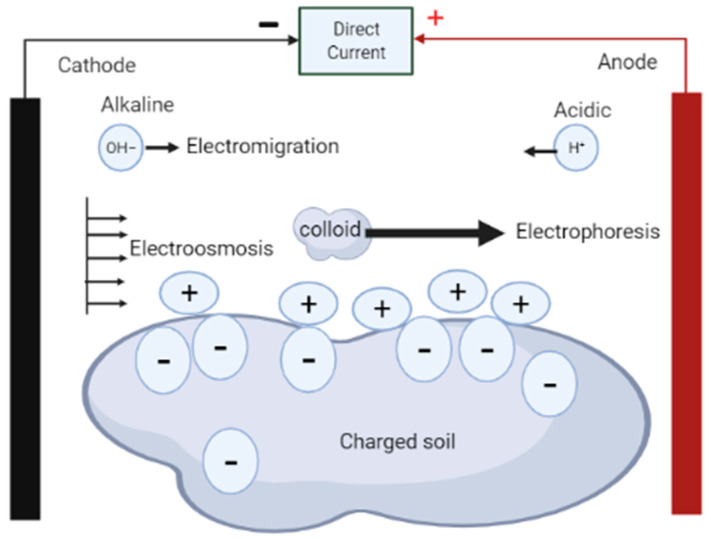
Fundamental transport mechanisms induced by the application of current.

As shown in Figure 1, the electrokinetic phenomenon is made up of five fundamental processes: electrolysis, electroosmosis, electrophoresis, diffusion, and electromigration [23,24]. In electrolysis, chemical reactions occur at the electrodes to decompose water into ions and gases [23]. Electroosmosis involves movement of pore liquids in the media due to the existence of the electrical double layer at the solid–water interface [23,24,29]; electrophoresis involves movement of charged colloids in the medium relative to the stationary fluid of the media [23], while diffusion is the distribution of compounds in the media from regions of higher concentrations to regions of lower concentrations [25]. Electromigration, on the other hand, is regarded as the main mechanism for electro-remediation and involves movement of charged ions to oppositely charged electrodes due to application of an electric field [23,29]. Acar et al. [28] argues that electrokinetic remediation is considered a technically feasible and cost-effective method of decontamination of contaminated soil because of the complementary combination of electrolysis, electroosmosis, electrophoresis, diffusion, and electromigration. In addition, the migration flux in the system enables transportation of contaminant species to where they can be removed from the matrix [28].

When land is degraded by organic pollutants due to introduction of petrochemical hydrocarbons (such as oil) in the soil, the contamination process is accompanied by creation of strong emulsions of soil solids, water, and oil after the pollution event [31]. Emulsifying compounds, such as organic acids, finely divided minerals, asphaltenes, waxes, and resins, found in petrochemicals aid in the creation of soil–water–oil emulsions and lowering the demulsification force, making it extremely difficult to separate the constituents [31,32]. Elektorowicz et al. [31] claim that electrokinetic remediation can effectively remove organic compounds from contaminated matrices by enhancing demulsification. It is reported that application of electrokinetics in soil contaminated with oil can increase demulsification by 200%, leading to separation of the constituents of the emulsion and enhanced recovery of oil from the matrix [31]. Table 1 shows some of the organic compounds that are commonly remediated by electrokinetics.

Application of current leads to electro-coalescence of small oil/water droplets into larger aggregates, forming a liquid phase that can be separated by electroosmosis [26,31]. The current also leads to transportation of charged colloidal particles by electrophoresis and movement of ions to oppositely charged electrodes [26,31]. Polar organic molecules, ionic micelles, colloidal electrolytes, and ionic metals are all transported by electromigration, but transportation of both organic and inorganic compounds is mainly facilitated by electroosmosis [47]. The disassociation rate of the constituents of the matrix being treated depends on their octanol/water partition coefficient and solubility [48]. Application of current in the electrokinetic system catalyses several chemical reactions that affect the remediation process [47]. These may include adsorption–desorption, dissolution–precipitation reactions, acid–alkaline reactions, and redox reactions [27,47,49,50]. Due to numerous and complex electrochemical and physico-chemical processes within the electrokinetic system, meticulous understanding of the following is required [9,51]:

Mass transfer in the electrolyte wells;

Distribution of the electric potential in the system; 

Adsorption of compounds onto colloids

Balance of the pore fluid per unit volume in a pore medium

Chemical speciation and transportation.

However, decomposition of water at the electrodes is one of the most important reactions that cannot be overlooked. The decomposition of water involves reduction reactions at the cathode (Equation (1)) and oxidation reactions at the anode (Equation (2)) [47,48,49,52,53,54,55].

(1) $$4H_{2}O+4e^{-}\to 2H_{2\left(g\right)}+4{\mathrm{OH}}_{\left(\mathrm{aq}\right)}^{-}$$

(2) $$2H_{2}O\to 4e^{-}+4H_{\left(\mathrm{aq}\right)}^{+}+O_{2\left(g\right)}$$

## 3. The Role of pH Distribution and Its Effect on Electrokinetic Remediation

As already elucidated, one of the most important reactions in an electrokinetic system is decomposition of water at the electrodes leading to formation of OH^−^ ions at the cathode and H^+^ ions at the anode. When current is applied, an alkaline front is formed at the cathode due to generation of OH^−^ ions, while an acidic front is formed at the anode as a result of generation of H^+^ ions [53]. These ions migrate towards each other to attain oppositely charged electrodes with H^+^ ions that are almost twice as mobile (1.75 times) as OH^−^ ions [47]. The movement of these ions makes the system more acidic due to the dominance of protons since H^+^ ions have a higher migration speed than OH^−^ ions. This is reversed when H^+^ ions meet OH^−^ ions to form water [53]. This means that electrokinetic cells have a highly dynamic pH that depends on generation and movement of the ions produced from decomposition of water if water is part of the matrix porewater [27,47,53,56].

To understand electrochemical processes during electrokinetic remediation, the behaviour of matrix compounds within widely varying pH values should be evaluated [28]. Production of ions as a result of electrolysis is a very important aspect in decontamination of contaminated media [56]. For example, H^+^ ions are reported to increase dissolution of contaminants and enhance desorption of pollutants from their absorbents, such as soil [56]. It is also claimed that acidic conditions in the system resulting from dominance of protons enhances electrochemical oxidation reactions [27,57]. However, at times, the ions produced in the system combine with compounds in the media to form complex compounds that could enhance or adversely affect the remediation process [26]. Giannis et al. [56] reported that generation of OH^−^ ions in media containing heavy metals could reduce decontamination efficiency by precipitating heavy metals in the cathode due to high pH. In general terms, high concentrations of ions in the electrokinetic cell result in a reduction in the electroosmotic flow to unmeasurable levels [28]. High concentrations of ions increase the thickness of the electrical double layer, confining the electroosmotic flow of the pore fluid to the periphery of the system/vessel [28]. Electroosmotic flow ultimately stops when the changes in the system composition result in the soil surface potential approaching zero or when the electroosmotic fluid flux equals the counteracting flux under the hydraulic gradient [28].

The convergence of the acid and alkaline fronts in the system may also affect removal of contaminants from soil [28,45,55,58]. This is due to focusing effects that emanate from precipitation of pollutants at the pH junction [28,45,55,58]. Resolving the impacts of focusing effects calls for pH control, use of ion exchange membranes, and/or use of conditioning or chelating agents [28,58,59]. Some of the commonly used agents are acetic acid, oxalate, citrate, and ammonia [28,58,59]. Yuan and Chiang [60] warn that control of the pH is inexorable if the target of effective removal of contaminants using an electrokinetic system is to be achieved. This is further validated by the fact that pH also affects the surface charge of surfaces, which, in turn, affects the zeta potential [22,57]. The relationship between pH and zeta potential (ς) as adopted from Park et al. [45] is shown in Equation (3) below.

(3) $$\varsigma \left(\mathrm{mV}\right)=38.6+281e^{-0.48pH}$$

### 3.1. Use of Ion Exchange Membranes

Ion exchange membranes are used to control the pH by controlling the movement of ions produced as a result of electrolysis. Cation exchange membranes prevent movement of anions through the membrane, while anion exchange membranes prevent movement of cations through the membrane [61]. An anion exchange membrane placed between the anode and the soil prevents movement of H^+^ ions produced due to electrolysis of the anolyte from migrating to the contaminated soil, while a cation exchange membrane placed between the cathode and soil prevents movement of OH^−^ ions produced from electrolysis of the catholyte into the contaminated soil [62]. By controlling migration of H^+^ ions and OH^−^ ions into the contaminated soil, ion exchange membranes are able to eliminate the need for additives that are required to control the pH in the electrochemical environment arising from movement of ions to parts where they may not be needed [63].

### 3.2. Electrode Conditioning for pH Control

To avoid contaminant precipitation and ensure that electroosmotic flow is not inhibited during remediation, pH control, especially at the cathode, is very important. Weak acids are often circulated at the cathode to neutralise OH^−^ ions produced during electrolysis of the fluids. Weak acids, such as acetic acid, hydrochloric acid, and citric acid, are commonly used for cathode conditioning [28,45,46,58]. However, use of some acids, such as chloric acid, poses health, safety, and environment (HSE) risks, such as generation of chlorine gas. Therefore, use of organic acids, such as acetic acid and citric acid, is preferred because they have a good acid/base buffer capacity, they are biodegradable and environmentally friendly, prevent formation of insoluble salts, and reduce the energy expenditure of the process by increasing or maintaining the electrical conductivity of the system [64].

### 3.3. Use of Chelants and Complexing Agents

Chelants are mainly applied to electrokinetic systems to increase the solubilisation of contaminants [65]. Chelation involves the use of a chelator or sequestering agent to form a separate bond between a single metal central ion and a multi-dentate/bi-dentate ligand. This leads to formation of chelate complexes due to the coordination of the chelator with the central metal ions at two sites or more [66]. Carboxylates, polyamines, industrial wastewaters, and organophosphonates, such as ethylenediaminetetraacetic acid (EDTA) and citric acid, have been used extensively in lab-scale studies [64,65,67]. Chelators also have the tendency to lower the zeta potential of soil particles, which leads to an increase in the electroosmotic flow rate of the electrokinetic system. To ensure that electrokinetic remediation is implemented in a sustainable and clean manner, nontoxic industrial waters containing mono-sodium and citric acid industrial wastewater can be used as chelators [68]. Some chelators, such as EDTA, are considered to be non-biodegradable and can lead to further contamination of the soil by creating stable complexes with natural soil minerals and are generally resistant to biodegradation, which renders treated soils intoxicated [67]. In general, selection of a chelator should, among other factors, include strong extraction strength, high affinity towards the contaminant, chelator recovery, cost-effectiveness, and low environmental toxicity [67,68].

Unlike chelators, complexing agents only form a single bond between the central metal ion and the complexing agent. Examples of commonly used complexing agents are lactic acid, acetic acid cyclodextrins, and ammonium acetate [68]. Complexing agents, such as acetic acid, can be used to control the pH of the system by creating an acid/base buffer where electrolysis products, such as OH- ions at the cathode, can be neutralised [67]. This can lead to a reduction in energy expenditure by reducing the rate of electrolysis at the cathode. Acetic acid and cyclodextrins are relatively cheap, non-toxic, and biodegradable [68].

### 3.4. Use of Reducing/Oxidising Agents

To implement electrokinetic remediation as a clean process, reducing/oxidising agents can be used to reduce the toxicity potency of the target contaminant or process by-products. If electrokinetic remediation is coupled with bioremediation, reducing/oxidising agents can stimulate bacterial growth and contaminant biodegradation by breaking down contaminants to less complex units that can easily be degraded by bacteria or provide an optimum oxygen supply that may be needed by aerobic biodegrading organisms. Injection of reducing/oxidising agents is commonly completed through the Fenton process to facilitate reactions between Fe^2+^ ions and H_2_O_2_ to degrade compounds, such as chlorophenols, phenols, and benzene, as shown in Equation (5) [66].

(4) $${\mathrm{Fe}}^{2+}+H_{2}O_{2}\to {\mathrm{Fe}}^{3+}+{\mathrm{OH}}^{•}+{\mathrm{OH}}^{-}$$

(5) $$\mathrm{RH}+{\mathrm{OH}}^{•}\to H_{2}O+R^{•}$$

(6) $$R^{•}+{\mathrm{Fe}}^{3+}\to {\mathrm{Fe}}^{2+}+\mathrm{products}$$

The Fenton process starts with the oxidation of Fe^2+^ ions to Fe^3+^ ions and the decomposition of H_2_O_2_ to form hydroxyl radicals (Equation (4)) [66]. In Equations (5) and (6), organic contaminants are oxidised and degraded by hydroxyl radicals [66]. Complete mineralization of the products can be achieved by further oxidation. Previous Fenton processes have mainly made use of ferric salts, heterogeneous catalysts, and metal ions as catalysts to incite the Fenton reaction mainly because they produce less toxic reaction by-products, are environmentally friendly, and are relatively cheap as compared to other catalysts [68]. The problem with Fenton processes is the need for acidic conditions (pH of 3–5) requiring pH control and adjustment [68].

## 4. Effects of Electrodes and Electrolytes in Contaminant Removal

The electrokinetic system is composed of an anode and a cathode as the electrode pair with the anolyte as the electrolyte nearest to the anode and the catholyte as the electrolyte nearest to the cathode [25,69]. Electrodes can be applied in different ways, such as conventional anode–cathode configuration, alternative anode and cathode approach (where a constant voltage is applied until the polarity is reversed after a specific period), two-anode technique (where an extra anode is introduced to produce hydrogen ions that can counter the alkaline front), and approaching anodes (where the anode is moved towards the cathode at different time intervals). In remediation of contaminated media, reactions at the electrodes and the spacing of electrodes have been found to affect the efficiency of contaminant removal [20]. For instance, in the variation of electrode spacing from 4 cm to 6 cm to 8 cm in the electrokinetic dewatering of oil sludge by Yang et al. [26], it was observed that the highest dewatering efficiency of 56.3% was achieved with the lowest spacing of 4 cm, while the highest oil recovery from the oil sludge was achieved with the highest electrode spacing of 8 cm. In the comparison of fixed and approaching anodes (continuous reduction in electrode spacing) for the removal of chromium contaminants from soil by Li et al. [59], it was concluded that approaching electrodes were the most efficient, with the highest removal of Cr (total) (35.96%) and Cr (VI) (92.50%). Li et al. [59] cited that, unlike fixed electrodes, approaching electrodes enhanced the current and decreased the soil pH, leading to higher contaminant removal. In the same vein, Zhang, et al. [70] reported that adoption of approaching electrodes to remove lead from contaminated soil achieved the highest removal efficiency of 83.8% and prevented precipitation of lead in soil and decreased the operational time and the energy consumed.

Electrokinetic remediation is supposedly also affected by voltage, which is highly varied by electrode voltage losses during electrochemical processes [20,26]. Materials with high electrochemical potential have high electrode–electrolyte interface losses and vice versa [26]. For example, in the comparison of iron and carbon materials as anodes, it was observed that voltage losses at the carbon anode amounted to +1.18 V, while the voltage losses at the iron electrode were −0.44 V [26]. These losses led to a tremendous increase in generation of heat in the system [26]. Besides affecting voltage, electrodes may introduce contaminant ions into the system that affect the decontamination process. This was observed in removing lead from contaminated soil using iron anodes, where the production of Fe^2+^ ions at the anode precipitated as Fe(OH)_2_ in the system, thereby affecting the process of lead removal [71].

Inert electrodes should, therefore, be used to prevent production of contaminant species at the electrodes [28]. It has previously been suggested that high-grade carbon should be used as the anode due to the acidic conditions associated with the surrounding environment, while low grade metals may be used as the cathode [28]. It is further suggested that complexing, processing, or conditioning fluids may be used where it is impossible to use high-grade carbon as the anode [28].

In the electrokinetic remediation of heavy metal contaminated kaolin, it was noted that electrodes may also have a significant effect on the rate of electroosmotic flow, which, in turn, affects the overall contaminant removal process [72]. Yuan, et al. [73] reported that use of carbon covered polyethylene terephthalate yarns (PEC-CNT) electrodes led to elevated removal of zinc, nickel, and cadmium as compared to graphite and Pt/Ti electrodes, citing that PEC-CNT increased the current and the electroosmotic flow, leading to improved heavy metal removal. It is acknowledged that, besides operation time, energy, electrolyte, and voltage, great emphasis has to be put on electrode configuration because it greatly affects the efficiency and cost of contaminant removal [72]. Hence, strategies, such as use of approaching electrodes, have been developed to decrease the pH when needed, increase electromigration, and save energy [55,70]. In fact, it has been shown that approaching electrodes can reduce the energy of the remediation process by 44% and time by 40%. Energy consumption per unit volume of the matrix treated can be calculated using Equation (7) [30,52,60,70,74].

(7) $$E_{u}=\frac{1}{V_{S}}\int VIdt$$

where *E_u_* is the energy calculated as kWh/m^3^, *V_S_* is the volume of the medium, such as soil, *V* is the voltage difference between the electrodes, and *I* is the electric current.

Electrolytes affect remediation according to how fast they decompose into ions [75]. The lower the concentration of ions generated in the system, the lower the current and vice versa [75]. For instance, Zhu et al. [75] reported that ammonia water as an electrolyte in the remediation of fluorine-contaminated soil led to generation of higher ion concentrations and current variations as opposed to when deionised water was used. It has been reported that high electrolyte concentration amidst low pore fluid pH can lead to a change in direction or reversal of electroosmotic flow by interfering with the polarity of the vessel surface [28].

Electrode selection is very important in reducing voltage losses in an electrokinetic system. Electrode materials with a high surface potential, such as carbon, are prone to voltage losses at the soil–electrode interface compared to low-surface-potential surfaces, such as steel. Highly corrosive electrodes should be used under controlled pH or should be coated with corrosion inhibitors to prevent rapid corrosion in acidic conditions. Inclusion of coatings on electrodes can produce hydrogen peroxide, chlorine, and high energy free radicals, such as $O_{2}^{-•}$, ${\mathrm{Cl}}_{2},$ and ${\mathrm{OH}}^{•}$, which can actively become involved in oxidation of pollutants.

Electrodes can also be chosen depending on whether there is a need for them to be actively involved in chemical reactions or not. For instance, active anodes (such as Pt, IrO_2_, graphite, RuO_2_, and carbon) and non-active anodes (such as PbO_2_, SnO_2_, and boron-doped diamond) can all be used to drive anodic oxidation of pollutants in an electrokinetic system. The interaction between the electrolytes and the electrodes leads to formation of °OH, as shown in Equation (8), where the electrode is denoted as MO_x_ [76]. In the presence of active electrodes at the anode, the °OH strongly adsorbs to the electrode to form a metal oxide (MOx + 1), as shown in Equation (9). Organic contaminants are either oxidised directly by the electrodes, as shown in Equations (10) and (11), or indirectly by high energy free radicals °OH (Equation (8)), H_2_O_2_ (Equation (12)), and O_3_ (Equation (13)) to form carbon dioxide [76]. Incorporation of electrodes that aid in the breakdown of organic pollutants can ensure complete mineralisation of the pollutants and reduce the longevity of the treatment process, hence enabling treatment cost reduction [76]. Production of free radicals, such as $O_{2}^{-•}$, ${\mathrm{Cl}}_{2},$ and ${\mathrm{OH}}^{•},$ should be avoided in bio-electrokinetic remediation, where microbes are partially utilized for the breakdown of the pollutant because they inhibit microbial growth [5].

(8) $${\mathrm{MO}}_{x}+H_{2}O\to {\mathrm{MO}}_{x}{(}^{\circ }\mathrm{OH})+H^{+}+e^{-}$$

(9) $${\mathrm{MO}}_{x}{(}^{\circ }\mathrm{OH})\to {\mathrm{MO}}_{x+1}+H^{+}+e^{-}$$

(10) $$R+{\mathrm{MO}}_{x+1}\to {\mathrm{MO}}_{x}+\mathrm{RO}$$

(11) $$2{\mathrm{MO}}_{x}{(}^{\circ }\mathrm{OH})\to 2{\mathrm{MO}}_{x}+H_{2}O_{2}$$

(12) $$2{\mathrm{MO}}_{x}{(}^{\circ }\mathrm{OH})\to 2{\mathrm{MO}}_{x}+H_{2}O_{2}$$

(13) $$3H_{2}O\to O_{3}+6H^{+}+6e^{-}$$

## 5. Electroosmosis and Its Effects on the Remediation Process

Electroosmosis is dependent on the surface charge of the matrix, the pore fluid dielectric constant, temperature, ionic concentration, and viscosity [22,47,53,77]. The relationship between electroosmotic flow and other factors is described by Helmholtz–Smoluchowski’s kinetic equation (Equation (14)), where EOF (m/s) is the electro-osmotic flux, E_x_ is the electric field, Z is the zeta potential of the soil, D is the dielectric constant, ε*_o_* is the vacuum permittivity, and μ is the fluid viscosity [19,74,78].

(14) $$\mathrm{EOF}=\frac{-D\varepsilon_{o}Z}{\mu }E_{x}$$

The direction of the electroosmotic flow depends on the soil’s zeta potential [45,79]. The surface charge of soil can either be temporary or permanent due to adsorption of ions (hydroxide and hydrogen ions) or isomorphic substitution, respectively [45,79]. At a high pH, the zeta potential is usually negative, forcing EOF towards the cathode, while, when the pH is low, the zeta potential is often positive, forcing EOF towards the anode [24,48,80]. The liquid phase may gravitate towards the anode on some occasions even when the matrix is negatively charged [26]. For instance, Yang et al. [26] observed the flow of oil and grease from contaminated sludge towards the anode much as the flow of water was towards the cathode. This unexpected EOF of grease and oil towards the anode instead of the cathode prevented reactions in the oil, grease, and hydroxides that would have led to formation of soap that would have adversely affected the sludge treatment process [26]. However, it has been reported that a decrease in pH leads to a direct reduction in EOF [27]. Since a low pH results from a high concentration of H^+^ ions, these ions impose a negative positive charge on the solid media, which reduces the EOF of fluids [27,81].

Use of chelating and conditioning agents is proposed as an effective technique to enhance and change the direction of EOF, which, in turn, leads to effective remediation [77,79,82,83,84]. Furthermore, these agents can be used at electrode compartments to control the pH, thereby enhancing contaminant removal, as already elucidated in Section 3 [80,82,85]. However, alkaline conditioning is said to be a better conditioning technique as compared to acidic conditioning if positive results are to be achieved [79,80].

As shown in Helmholtz–Smoluchowski’s kinetic equation, EOF can be affected by the viscosity and the molecular size of the pore fluids [26,78]. A previous study by Yang et al. [26] reported accumulation and stagnation of oil in the medium compartment of an electrokinetic cell until the EOF of water was reduced, allowing subsequent flow of oil towards the cathode compartment. This was attributed to the differences in the molecular size of water and oil molecules. Water (small molecules) dominated the EOF through the filter; the flow of oil (larger molecules) was only observed when the EOF of oil had reduced [26]. Gidudu and Chirwa [86] also reported accumulation of oil at the cathode–medium interface due to the dominance of water in membrane pores as it moved from the anode towards the cathode.

## 6. Electromigration and Its Effects on the Remediation Process

The separation rate of phases and contaminants in contaminated soil depends on their polarity [31]. Disassociation of compounds into ions is mainly reliant on their dielectric constants, while electromigration is dependent upon the rate of disassociation [87]. For instance, the dielectric constant of water is three times the dielectric constant of non-aqueous substances, which means that, if a large number of ions were to participate in the electrolysis of water, higher electromigration and current flow would be observed in water as compared to non-aqueous substances, such as cosolvents [87]. However, high concentrations of ions, especially H^+^ ions in the system, affect electroosmosis when they impose a negative positive charge on solid media [27,81]. This explains why transportation and concentration of ions are as important as EOF during electrokinetic remediation [28].

In remediation of soil contaminated with hydrocarbons in the presence of pore water, the organic aqueous interfaces of contaminants usually take up a negative charge due to absorption of hydroxyl ions produced from the cathode [31]. Much as organic compounds, such as aromatics and aliphatics found in petrochemicals, are hydrophobic, after obtaining a net negative charge from the hydroxyl ions, they can effectively be removed from the media by electromigration [31]. Electromigration is affected by the presence of competitive ions, the initial concentration of the specific ions in the medium, ionic mobility, current density, pore water, grain size, applied electric potential, pH gradient, conductivity, and porosity of the solid medium [22,47]. It is argued that the force (F) applied to induce the movement of ions in an electrokinetic system is a product of the charge of ionic species (Z_i_), elementary charge (e =1.6 × 10^−19^ C), and voltage gradient (∇Vin V/cm), as shown in Equation (15) [57].

(15) $$F=Z_{i}e\times \nabla V$$

Current flow in an electrokinetic system is dependent on the conductivity of the soil, water content, and the voltage applied [27]. The current often rises in the initial stages of the remediation process depending on the conductivity and resistance of the soil [27,48]. The increase in current during the initial stages of remediation is attributed to the high concentration of ions and their movement in the system by electromigration [88]. This is often observed until equilibrium is reached, when ions in the system react with compounds in the system, leading to current decrease [88]. The reduction in current is also related to the reduction in the concentration of mobile ions or the increase in the resistance of the media, also referred to as resistance polarisation [48,55].

## 7. Demulsification of Emulsions by Application of an Electric Current

Emulsions are a mixture of two or more liquids that are naturally immiscible. Emulsions exist as a colloidal system of small droplets, with dimensions in the range of 1 nm to 1 μm in a continuous phase [89]. Depending on which of the liquids is the continuous phase, water and oil emulsions may exist as either W/O or O/W emulsions [89]. One of the largest problems associated with remediation of solid media contaminated with hydrocarbons is the difficulty to separate strong and stable emulsions created from oil, water, and solids [31,32]. The stability of emulsions in contaminated media during electrokinetic remediation is dependent on viscosity, interfacial tension, wettability, electrolyte, electrical potential, agitation, hydrophile–lipophile balance, phase volume ratio, and temperature [31]. Application of current leads to electro-demulsification of compounds in the matrices, starting with the breakdown of colloidal particles that are then transported vertically [31]. At the same time, the pore fluids are transported horizontally [31]. Movement of colloids by electrophoresis and movement of pore fluids by electroosmosis are important processes during remediation [31,47]. The movement of colloids in the electrokinetic system is because of their polarity, which enables them to be transported by electromigration to oppositely charged electrodes [23]. The colloids often aggregate when they are transported to the electrodes, with the rate of aggregation dependent on the electrical potential applied [31]. Depending on the intensity of the electric field, the coagulation could be slow or fast [31]. Fast coagulation creates aggregates of loose particles, while slow coagulation creates aggregates of compact particles [31].

Electro-demulsification depends on electrical potential since the rate of demulsification is influenced by the intensity of the voltage applied [31]. For instance, in the variation of electrical potential from 0.5 to 1.5 V/cm in the optimisation of an electrokinetic cell for phase separation, Elektorowicz and Habibi [90] observed that the variation in voltage did not have any significant effect in terms of oil recovery and water recovery. Yang et al. [26] reported that an increase in voltage from 10 V to 20 V led to an increase in the dewatering of sludge, but a further increase to 30 V did not have any significant impact on the process. Contrary to this, Gidudu and Chirwa [5] reported that an increase in voltage from 10 V to 30 V significantly increased the removal of hydrocarbons from the soil from 66% to 74%. 

The rate of demulsification can be determined using Equation (16), where H_0_ is the height of the emulsion before the experiment and H is the height of the emulsion after the experiment, K_d_ is the overall demulsification rate constant, and t is the duration of the experiment [90].

(16) $$\frac{H}{H_{0}}=\exp\left(-k_{d}t\right)$$

## 8. Combination of Electrokinetic Remediation with Other Technologies

**Figure 2:**
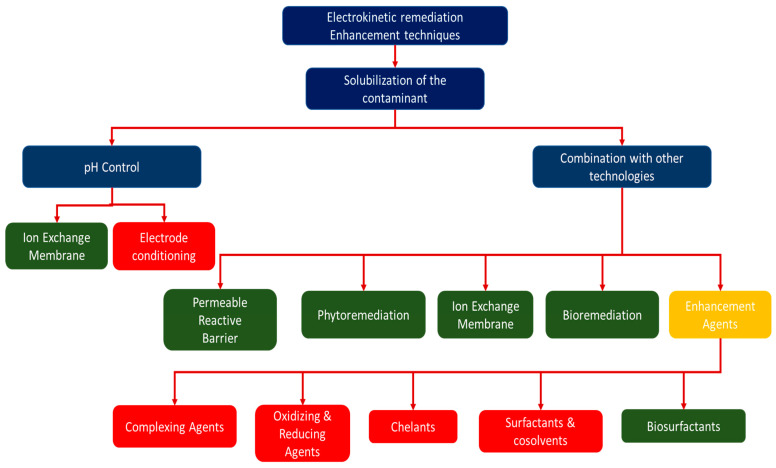
Technologies/techniques combined with electrokinetic remediation. Green represents the clean techniques, while red represents techniques that are regarded as environmentally unsound.

Figure 2 shows technologies/techniques that have been combined with electrokinetic remediation to make electrokinesis more efficient. Some of these technologies can be categorised as clean technologies because they are environmentally friendly and have a low footprint (marked as green in Figure 2), but some may have adverse environmental effects (marked in red in Figure 2). Combination of electrokinetic remediation with other remediation technologies, such as permeable reactive barriers, oxidation, and application of chemicals, has previously been suggested to prevent the effect of pH variation and increase the efficiency of the electrokinetics [47,50]. Technologies such as the Lasagna permeable reactive barrier have previously been combined with electrokinetic technology to allow in situ remediations of contaminated soils [91,92,93]. In previous studies, chelators were also combined with electrokinetics to enhance the removal of pollutants and prevent precipitation in the decontamination of concrete, clay soils, and wastewater treatment sludge [57,94,95]. To avoid use of chelators that can lead to further contamination of the system, the use of polarity exchange has been studied and suggested as an effective method to prevent precipitation of contaminants in an electrokinetic cell [50,78,96]. It should, however, be noted that combination of electrokinetic remediation with other methods can increase the costs of remediation extensively [47,50]; indeed, Cang et al. [85] claim that the efficiency of removing contaminants is usually very low without enhancements.

### 8.1. Surfactants in Electrokinetic Remediation

Organic pollutants, especially those that pose a threat to the environment, are non-ionic, have non-ionisable molecules, and are insoluble in water [19,47]. Boulakradeche, Akretche, Cameselle, and Hamidi [19] argue that the low solubility and hydrophobic properties of organic pollutants make it difficult to remediate contaminated soil by only relying on electroosmosis and electromigration. It has previously been underscored that, because of the recalcitrant properties of organic pollutants, high solubilisation of the contaminant has to be attained by application of surfactants, which can simultaneously be combined with electroosmosis to obtain effective remediation [47]. Otherwise, electrokinetic remediation may have to be combined with other technologies to remove organic contaminants efficiently [47]. Surfactants are, therefore, applied to enhance the solubilisation of the pollutant and attain increased mobility of the pollutants [22,74].

The hydrophobic tail of a surfactant allows it to gravitate towards the hydrophobic molecules of hydrocarbons, while the hydrophilic head of the surfactant enables it to easily solubilise in water [46]. These properties allow surfactants to reduce surface tension, obtain micellization, solubilisation of contaminants, and increase adsorption of the compounds [46,74]. Surfactants alter the surface properties of the contaminated matrix, leading to enhanced mobility of the contaminant by electromigration, electrophoresis, and electroosmosis [46,74,97].

The effectiveness of the surfactants during electrokinetic remediation mainly depends on the properties of the matrix and the properties of the surfactants [46]. It is claimed that neutral surfactants should be chosen over cationic and anionic surfactants because neutral surfactants can be transported through the system by electroosmosis. Ionic surfactants should be avoided since they interact with the matrix, leading to a decline in the remediation efficiency [19,74]. Much as neutral surfactants may be preferred in most cases, they can also interact with cations in the electrokinetic system if they bond with hydrogen ions [74]. Polarisation of neutral surfactants can lead to a decrease in EOF [74].

On the other hand, anionic surfactants are preferred to cationic surfactants on the basis that anionic surfactants often enhance electroosmosis and electromigration by introducing a negative zeta potential on the matrix as opposed to cationic surfactants [97]. Anionic surfactants also have high solubilisation properties and are highly biodegradable compared to cationic surfactants when they end up in the environment [46]. It should, however, be noted that anionic surfactants may affect the remediation process by moving in the direction opposite to the EOF if the flow is from the anode towards the cathode [19,74]. The rate of removal of the contaminants using surfactants highly depends on the binding capacity of the pollutant with the surfactant micelle [60]. This is probably why, in the past, researchers focused on the use of anionic surfactants to remove cationic pollutants and vice versa [60].

Park et al. [45] reported that application of a non-ionic surfactant in the pore fluid enhanced removal of lubricant oil to attain the highest removal efficiency of 55.4% at 1.0 V/cm. This was attributed to transportation of the surfactant throughout the system by electroosmosis [45]. In another study, it was observed that addition of an amphoteric surfactant (C_12_-C_14_-alkyl-dimethyl-betain) did not improve the hydrocarbon removal process [92]; without surfactants, 43% removal of hydrocarbons and 63% removal of water from the contaminated media were achieved. The addition of the surfactant slightly increased the hydrocarbon removal to 50% but decreased the water removal to 60% [90].

**Figure 3:**
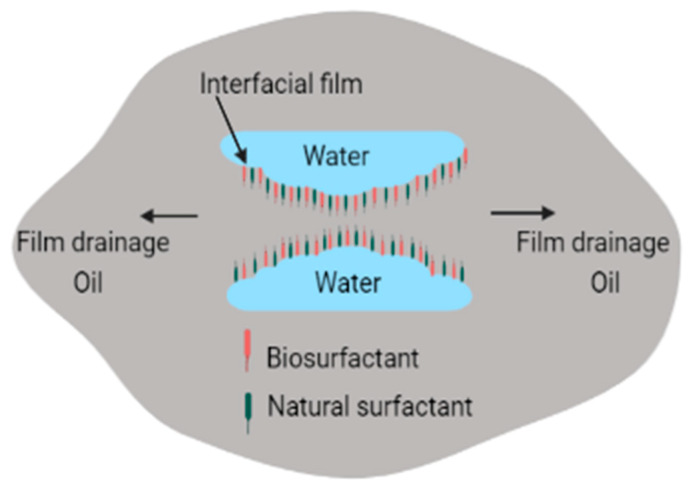
Demulsification of emulsions by biosurfactants.

Numerous types of surfactants have been used in electrokinetic remediation, as shown in Table 1. The greatest disadvantage associated with addition of chemical surfactants, processing fluids, conditioning agents, and chelating agents is that these combine with compounds in the matrix to produce complex and dangerous compounds that may threaten humans and the environment [97]. Use of surfactants for enhanced oil recovery and environmental remediation is fast and efficient [71,98]. It can also be used to treat large volumes of contaminated media, but chemical surfactants are costly and are toxic to the environment [71,98]. To overcome the problem of toxicity, use of biosurfactants has been proposed as a potential replacement of synthetic surfactants because of their lower toxicity, high biodegradability, high diversity, high demulsification potential, and selectivity [99]. Biosurfactants can also be used effectively in varying salinity, pH, and temperature [100,101,102,103]. In addition to electro-demulsification obtained by applying current, biosurfactants can enhance demulsification by reacting with emulsifiers found in petrochemicals. This occurs when biosurfactants adsorb on oil–water interfaces, leading to elimination of the thin films between oil–water interfaces, as shown in Figure 3 [89].

**Figure 4:**
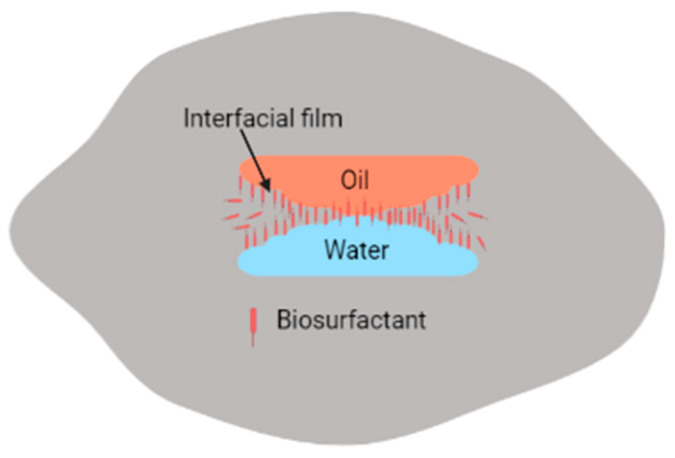
Emulsification of emulsions by biosurfactants.

Adsorption of biosurfactants at oil–water interfaces leads to increased coalescence of distinct phases of water and oil (containing hydrocarbon contaminants), which can then be separated electrokinetically [45,90]. However, similar to synthetic surfactants, biosurfactants may promote, inhibit, or have no significant effect on remediation processes [104]. For instance, if high-molecular-weight biosurfactants are used instead of low biosurfactants, emulsification of water, soil, and hydrocarbons occurs instead, as shown in Figure 4. The main difference between high-molecular-weight biosurfactants and low-molecular-weight biosurfactants is that high-molecular-weight biosurfactants prevent the coalescence of oil droplets in O/W or W/O emulsions since they have the ability to bind to the oil droplet surfaces, while the low-molecular-weight biosurfactants lower the surface tension and interfacial tension between oil–water droplets [5,86].

### 8.2. Combination of Electrokinetic Remediation with Bioremediation or Pytoremediation

Very few studies have been conducted to determine the effect of electrochemical processes on the enzyme activity, growth, survival, and movement of microorganisms within an electrokinetic system [105,106,107]. A few of the studies that have been completed have reported that microorganisms are affected mainly by electroosmosis and electrophoresis [108,109]. Microorganisms are affected because they are transported by electroosmosis and electrophoresis [78,108]. Other studies have reported that the electro-halo-thermal environment within an electrokinetic cell can kill bacteria due to application of an electric field and changes in pH and temperature [109,110].

For instance, in the study of the impact of electrokinetic remediation on microbial communities within pentachlorophenol-contaminated soil, electrochemical processes led to a reduction in bacteria by 17% and fungi by 30% [106]. In the study of the effect of electrokinetic remediation on indigenous microbes in contaminated soil, it was observed that application of current led to a decrease in soil microbial count, noting that the highest counts were observed at the area around the anode with 229 CFU g^−1^ soil, while the lowest count was at the area around the cathode with 48 CFU g^−1^ soil. In the remediation of soil contaminated by copper and zinc using a combination of electrokinetics and bioleaching, it was discovered that, for the growth of bacteria to aid in the process, pH was a major factor for consideration to have an efficient process [111]. Application of 0.63 mA cm^−2^ led to degradation efficiencies of 23.2% and 26.8% at a pH between 7 and 8 for light hydrocarbons (C_10_-C_16_), whereas, at a pH of 1–3, degradation efficiencies of 16.0% and 18.9% were obtained [109]. The death of bacteria during degradation was attributed to a reduction in bioavailability of the nutrients needed to support bacterial growth and the destruction of the functionality of the cell membrane [109].

Much as a high electric field may have detrimental effects, it is highlighted that application of a low electric field that does not exceed 10 mA in the system can result in high substrate utilisation and biodegradation, leading to high bacterial growth [106,109]. Electrochemical processes, such as electrolysis of water leading to production of oxygen and hydrogen together with the EOF of substrates, leads to an increased uptake of oxygen and the substrate by the microbes [106,109,112].

Chelating agents and conditioning and processing fluids, together with the electrolytes used, are likely to be detrimental to microbial growth [110]. Most of the conditioning agents used, such as ethylene diamine disuccinate, citric acid, ethylenediaminetetraacetic acid, and acetic acid, are toxic to the bacteria either because of their acidity or alkalinity properties [52,110,113].

Recently, researchers have tried combining electrokinetic remediation and phytoremediation to remove contaminants from soil [114,115]. In pyto-electrochemical remediation or electrochemical-assisted phytoremediation, plants are introduced in the presence of an electric field due to their metabolic structure, which allows them to extract contaminants from the soil [116]. The electric field applied assists in breaking down and moving the contaminants closer to where they can be extracted from the soil by plants [117]. The use of plants to enhance contaminant removal is a clean and sustainable alternative to the addition of chelating and conditioning agents to media during the treatment process because of their associated toxicity [118]. However, the problem associated with phytoremediation is that plants may not necessarily mineralize the contaminants but may rather bioaccumulate them, which requires post-treatment and meticulous disposal [115].

## 9. Future Prospects: Insights into Electrokinetic Remediation

### 9.1. Combination of Electrokinetic Remediation with Bioremediation Andbiosurfactants

To achieve field-scale production of biosurfactants for in situ bioremediation, biostimulation and bioaugmentation may have to be adopted. Biostimulation involves addition of nutrients to the media to enhance bacterial growth and contaminant degradation, while bioaugmentation involves addition of precultured microbes that can degrade to enhance degradation [119,120]. Much as bioremediation supported by biostimulation and bioaugmentation are extensively used, very few studies have been conducted to correlate adaption of these methods for biosurfactant production to enhance in situ bioremediation of contaminants in soil.

Ángeles and Refugio [121] studied the in situ production of biosurfactants and hydrocarbon removal by *Pseudomonas putida* CB-100 in bioaugmented and biostimulated oil-contaminated soil. In this research, biosurfactants were produced in biostimulated soil, with the highest biosurfactant yield of 1.88 ± 0.06 mg/kg and 1.97 ± 0.19 for irradiated soil and non-irradiated soil, respectively. The combination of biostimulation and bioaugmentation using *Pseudomonas putida* CB-100 led to a yield of 2.25 ± 0.21 mg/kg of biosurfactants in irradiated soil, while a yield of 1.7 ± 0.03 mg/kg of biosurfactants was obtained in non-irradiated soil. The highest degradation of total petroleum hydrocarbons was observed under combined biostimulation and bioaugmentation for treatment of both irradiated and non-irradiated soil [121].

In other studies, conducted to evaluate in situ production of biosurfactants by *Bacillus* strains injected into a limestone petroleum reservoir for oil recovery, it was reported that an average biosurfactant concentration of 90 mg/L was detected in the wells where either only nutrients were applied or both nutrients and bacteria were inoculated. In experiments involving biostimulation and bioaugmentation, a biosurfactant concentration of 350 mg/L was detected [122,123]. Zhao, et al. [124] reported that *Bacillus amyloliquefaciens* 702 and *Pseudomonas aeruginosa 709* produced 1582.4 mg/L and 8237.5 mg/L of biosurfactant under diverse conditions to enable enhanced oil recovery from an oil reservoir. In inoculation of *Bacillus licheniformis* RS-1 and *Bacillus subtilis subsp. subtilis spizizenii* NRRL B-23049 strains oil wells stimulated with nutrients for enhanced oil recovery, lipopeptide biosurfactant concentrations of 20 and 28 mg/L in the two wells were detected [123].

Gidudu and Chirwa [5] combined electrochemical remediation, application of biosurfactants, and bioremediation to achieve 74% removal of the pollutants in 240 h using a voltage of 30 V and a biosurfactant concentration of 28 g/L. In another study, the biosurfactant concentration was varied between 28 g/L, 56 g/L, and 84 g/L in the decontamination of petrochemical contaminated soil. It was then observed that the highest carbon removal was achieved when 84 g/L of biosurfactants were used, indicating that the addition of biosurfactants improved the efficiency of the remediation process [86]. To encourage field-scale applications of electrokinetics, some researchers have previously evaluated the potential of producing biosurfactants in situ and discovered that the bacteria inoculated produced biosurfactants within an electrochemical environment [125]. However, it was suggested that an intermittent or low current should be applied to protect bacteria from cell membrane destruction, citing that the highest yield of biosurfactants was only generated when the lowest current of 0.5 A was applied in that study.

It is argued that, unlike conventional remediation methods, electrokinetic remediation is efficient in soil with low permeability and can be used in railway soils and residential areas where it could be difficult to excavate. In situ electrokinetic remediation is possible, and it can simultaneously remove inorganic and organic contaminants [8]. Some of the field-scale applications have involved a pilot scale of electrokinetic remediation combined with solar as the energy source for the removal of Cd, Cu, and Pb from a 10 m × 30 m × 0.5 m polluted area [8]. Other field-scale studies have involved application of 48 V of voltage supplied through power transmission inverted to direct current to remove Pb and Cd [8]. Chung [126] also installed an electrokinetic system coupled with a permeable reactive pile in excavated landfill soil contaminated with Cu; effective removal of copper from in situ and sorted soils using the electrokinetic reactive pile system was reported.

Electrokinetic remediation can also be applied ex situ where solid contaminated media is transported to constructed electrokinetic treatment plants [127]. In the ex situ treatment of Pb contaminated soil in an electrokinetic remediation prototype plant built in Livorno, Italy, Masi et al. [128] demonstrated that solid contaminated media could successively be remediated ex situ at a plant that has access to all the necessary infrastructure, such as the energy source. The plant consisted of an ex situ treatment basin equipped with electrode wells arranged on a rectangular grid and connected to an electrolyte management system for catholyte and anolyte pH control.

Other commercial companies and organisations, such as the US Army Environmental Agency, ElectrosorbTM, and Electro-KleanTM electrical separation, operate at field scale to remediate contaminated soils. Much as electrokinetic remediation of soil in field-scale applications seemed farfetched decades ago, it is slowly advancing into field-scale applications currently [129]. To achieve electrokinetic remediation of soils on site, wells are excavated into contaminated media to accommodate electrode wells and electrodes. Current can be applied across the electrode pair to trigger the movement of the liquid phase and electromigration of contaminants in the soil. Similar to laboratory-scale findings, the pH drops at the anode and rises at the cathode, followed by movement of acidic and alkaline fronts [25,26]. To enhance the process, processing fluids, such as gallic acids, humic acids, and acetic acids, are applied to control the pH at the electrodes, enhance the migration of ions, and increase the solubilisation of the contaminants [25,26].

However, it should be noted that application of electrokinetic remediation both at the lab scale and field scale is very specific based on the type of soil, site, voltage applied, enhancements used, and the concentration of the contaminant, among other factors. These factors affect the mass transfer in the electrolyte wells, distribution of the electric potential in the system, adsorption of compounds onto colloids, balance of the pore fluid per unit volume in a pore medium, and chemical speciation and transportation [9,51]. These factors may influence electrolysis, electroosmosis, electrophoresis, and electromigration, which are then used to assess the cost of efficient remediation, feasibility, and practicability of the application of electrokinetic remediation at every specific site as these conditions would vary from site to site [130].

### 9.2. Energy Saving and Alternative Energy Sources in Electrokinetic Remediation

Electrokinetic remediation is based on application of current. Therefore, energy consumption is at the core of this technology. Much as the technology is generally sustainable in different ways, use of energy taints the sustainability of the technology in the general view of things, hence why integration of sustainable energy sources with electrochemical remediation should be considered inherently to meet the sustainable demands of the day. In an electrokinetic system, electrical energy is mainly associated with electromigration, electrophoresis, electrolysis, and ohmic losses of the system. Energy may also be needed to operate pumps needed to facilitate the flow of process fluids and any other additives. Alshawabkeh, et al. [127] claim that the total cost of energy represents 10–15% of the total cost and 25% of the total operating costs. Previous researchers have reported a reduction in energy expenditure based on modification of the electrode arrangement configurations and a reduction in the number of electrodes [131]. Use of pulsed electric fields where a switch off time is used between an on-pulse and the following pulse has been applied previously to reduce the energy budget without compromising the speed of remediation [21]. An energy saving of over 42% compared to the conventional electrokinetic configuration has been reported during application of pulse electric fields [21].

Other researchers have reported injection of ionic additives into the electrokinetic system to reduce energy consumption by increasing the ionic strength and conductivity of the system [21]. Fu, et al. [132] reported that addition of citric acid during the treatment of Cr reduced the energy consumption of the remediation process. Gidudu and Chirwa [133] also reported that addition of biosurfactants and approaching electrodes in an electrochemical system during the removal of petrochemical hydrocarbons reduced the energy budget by reducing the remediation time needed to obtain satisfactory decontamination. It was also acknowledged in another study that addition of biosurfactants and reduction in electrode distance led to a decrease in the energy expenditure by accelerating the decontamination process [5]. Use of renewable energy has also been recognized as a potential solution for achieving sustainability of the electrokinetic remediation process [105].

## 10. Conclusions

Much as electrokinetic remediation is a promising remediation technology, it is clear that more field-scale studies need to be conducted to understand the extensive impacts of voltage, electrodes, electrolytes, and surfactants on the remediation process. It is imperative that pH is controlled to avoid the focusing effect that may lead to precipitation of contaminants. Electrode configurations, such as approaching electrodes and application of surfactants, can enhance the process and reduce the energy expenditure of the remediation process. Since electrodes can corrode during the remediation process, it is important to consider inert electrodes to prevent deposition of toxic compounds during remediation. Furthermore, non-active electrodes, such as boron-doped diamonds, SnO_2_, and PbO_2_, can be used to enhance oxidation of organic pollutants. Ionic electrolytes can also be used to increase the ionic strength of the system while preventing the dehydration of soil that may result from electroosmotic flow of fluids. To attain sustainability of the remediation process, process additives, such as biosurfactants, can be used as a replacement for synthetic surfactants since they are environmentally friendly. Furthermore, cleaner sources of energy, such as solar and wind energy, can be adopted to fulfil the high energy needs of the remediation process where high voltages may be required to achieve effective decontamination.

## Figures and Tables

**Table 1:** Pollutants removed from soil by electrokinetic remediation.

Pollutant	Cathode-Anode	Electrolyte	Voltage /Current	Mass of Soil Treated (kg)	Surfactant type	Biosurfactant Concentration (g/L)	%Removal Efficiency	Reference
TPHs	Graphite-Graphite	Deionized water	30 V	2	Rhamnolipid	28.00	68.92	Gidudu and Chirwa [5]
TPHs	Graphite-Graphite	Deionized water	30 V	2	Rhamnolipid	28.00	74.77	Gidudu and Chirwa [5]
TPHs	Graphite-Graphite	Deionized water	10 V	2	Rhamnolipid	28.00	73.34	Gidudu and Chirwa [5]
TPHs	Graphite-Graphite	Deionized water	10 V	2	Rhamnolipid	28.00	66.45	Gidudu and Chirwa [5]
Diesel	BDD, Ti	Deionized water	30 mA cm^−2^	0.135	SDS	0.55–2.50	59–89	Saichek and Reddy [33]
PAH	BDD, DSA, stainless steel	Na_2_SO_4_	30 mA cm^−2^	0.1	cationic surfactant	2.50	100	de Melo Henrique, et al. [34]
Petroleum	BDD	Na_2_SO_4_	30 mA cm^−2^	0.01	SDS	0.10–50	92	Liu, et al. [35]
Diesel	BDD-carbon-felt	Na_2_SO_4_	30 mA cm^−2^	0.01	Tween 80	6.30–8.25	73–83	Liu, Oturan, Zhang and Oturan [35]
Phenanthrene	BDD- DSA (carbon felt)	Na_2_SO_4_	0.5–2 A	0.003	Hydroxypropyl-beta-cyclodextrin	1.60	58–99	Mousset, et al. [36]
TPH	BDD-carbon felt	Na_2_SO_4_	10–100 mA cm^−2^	15	Tween 80	11.00	100	Huguenot, et al. [37]
Crude oil	Graphite-Graphite	Na_2_SO_4_	1 V/cm	1	SDS	1.07	9.35	Li and Jiang [38]
Crude oil	Graphite-Graphite	Na_2_SO_4_	1 V/cm	1	rhamnolipid	1.07	14.06	Li and Jiang [38]
Crude oil	Graphite-Graphite	Na_2_SO_4_	1 V/cm	1	Tween 80	1.07	18.05	Li and Jiang [38]
DDT	Titanium-Titanium	Deionized water/CaCl_2_	20 V	0.5	Sodium dodecyl benzenesulfonate (SDBS)	7.50	13	Karagunduz, et al. [39]
Naphthalene	Graphite-Graphite	NaNO_3_	40 V	0.026	carboxymethyl-g-cyclodextrin	2.00	83	Jiradecha, et al. [40]
2,4-dinitrotoluene	Graphite-Graphite	NaNO_3_	40 V	0.026	carboxymethyl-g-cyclodextrin	2.00	89	Jiradecha et al. [40]
Ethylbenzene	Graphite-Graphite	processing fluid	2 V/cm	0.25	SDS and Pannox 10	5.03	98	Yuan and Weng [41]
Gasoil	Graphite-Graphite	Citric acid	30 V	4.3–4.5	Pannox 10, Citric acid	2.00	87	Gonzini, et al. [42]
Chlorobenzene and trichloroethylene	Graphite-Graphite	1-hydroxyethylidenediphosphonic	2 V/cm	4.3–4.5	Triton X-100, OS-20ALM	0.96	85	Kolosov, et al. [43]
Phenanthrene	Unspecified	Deionized water	12 V	0.33	Triton X-100 rhamnolipid	0.36	30	Chang, et al. [44]
Phenanthrene	Graphite-Graphite	Deionized water	2.0 V/cm	4	Igepal CA-720	23.50	90	Saichek and Reddy [33]
Lubricant oil	Carbon plate -Pt-coated titanium	HNO_3_	2 V/cm	Not specified	Tergitol	4.00	45	Park, et al. [45]
PAHs	Stainless steel- Stainless steel	NaCl	4.3 V/m	0.02	Tween 80	0.004	30	Park, Lee, Yang, Kim and Baek [45]
PAHs	Graphite-Graphite	Na_2_SO_4_	30 V	0.2	Tween 80	3.00	40	Alcántara, et al. [46]

## Data Availability

Not applicable.
